# Supplementary material for: Copy number variants implicate cardiac function and development pathways in earthquake-induced stress cardiomyopathy
Source: Sci Rep. 2018 May 15;8:7548. doi: 10.1038/s41598-018-25827-5 (PMC5954162; doi:10.1038/s41598-018-25827-5)
Supplement: Supplementary file 1 — Supplementary information [file 41598_2018_25827_MOESM1_ESM.pdf]

Copy number variants implicate cardiac function and development pathways in earthquake-induced stress cardiomyopathy

Authors

Cameron J. Lacey, Kit Doudney, Paul G. Bridgman, Peter M. George, Roger T. Mulder, Julie J. Zarifeh, Bridget Kimber, Murray J. Cadzow, Michael A. Black, Tony R. Merriman, Klaus Lehnert, Vivienne M Bickley, John F. Pearson, Vicky A. Cameron, Martin A. Kennedy.

Supplementary information

Supplementary details:

Analysis of candidate variants and genes identified in WES data p2
Heart-healthy control cohort p2
The Cardio-MetaboChip p3
ArrayCGH platforms used p3

Supplementary Figure S1: CNV interpretation algorithm p4

Supplementary Table S1: Complete CNV detection list of EqSCM cases in this study p5

Supplementary Table S2: Exome read count and coverage metrics p16

Supplementary details

Analysis of candidate variants and genes identified in WES data:

The chosen genotype prevalence cut off of 4/24 in the case cohort was an empirical threshold derived by iterative analysis. For example, when we filtered out all variants present at >3% MAF in 1000G, and required that variants be present in 4/24 samples, 131 variants remained on the list. Requiring the genotype to occur in a higher or lower proportion of cases resulted in respectively too few or too many variants for analysis.

The chosen MAF cut-off of >3% was also determined empirically, but reflected a desire to be inclusive of relatively common mutations or rare SNPs that might be relevant. We reasoned that with the strong environmental precipitant (exposure to a devastating series of earthquakes), and the requirement for post-menopausal status, an underlying predisposing genetic factor could be relatively common, in contrast to Mendelian diseases where the MAF cut-offs are typically much lower. Setting the MAF cut-off lower than 3% in our study resulted in very few candidate variants.

This list of genes was examined initially by a consideration of published gene function and expression data, predicted severity of the variant, as well as manual inspection of the BAM files to examine the context of the putative variant. Many of the variants proved to be artefacts of either the reference database (such as the minor allele being listed as a reference allele), or alignment issues, such as in repetitive sequence regions. We evaluated a handful of variants by Sanger sequencing on EQ-SCM cases in parallel with controls, and in all cases, the putative variants were excluded from further consideration (in most cases because they proved not to be present in the DNA).

Heart-Healthy Control Cohort:

The controls used in the Cardio-Metabochip analyses were a subset of the Canterbury Healthy Volunteers Cohort (total n>3,400), who were randomly selected from Canterbury electoral rolls, age- and gender-matched to existing patient cohorts (Ellis et al, 2011). They have no personal history of diagnosed cardiovascular disease at the time of recruitment, as indicated by a data search of the NZ Health Information Service (NZHIS) and a questionnaire confirming the absence of cardiovascular symptoms. Recruitment for this study commenced in January 2002 and is ongoing. Anthropometric measures, information on personal health, family heart history, heart disease risk factors and clinical characteristics are recorded and blood and DNA sampled at recruitment. Clinical events are documented from the NZHIS (median follow-up >5 years). (Ethics approval CTY/01/05/062).

Ellis, K. L. et al. Genomic risk variants at 1p13.3, 1q41, and 3q22.3 are associated with subsequent cardiovascular outcomes in healthy controls and in established coronary artery disease. Circ Cardiovasc Genet 4, 636-646, doi:10.1161/CIRCGENETICS.111.960336 (2011).

The Cardio-MetaboChip:

The custom Illumina iSelect Cardio-MetaboChip genotyping array (Voight et al., 2012) was designed to test ~200,000 SNPs (single gene polymorphisms), identified through genome-wide meta-analyses for metabolic, atherosclerotic and cardiovascular diseases and traits. The Cardio-MetaboChip was designed by representatives of the following GWAS meta-analysis Consortia: CARDIoGRAM (coronary artery disease), DIAGRAM (type 2 diabetes), GIANT (height and weight), MAGIC (glycemic traits), LIPIDS (lipids), ICBP-GWAS (blood pressure), and QT-IGC (QT interval). The traits covered by the panel of genetic variants on the chip include myocardial infarction (MI) and CHD, type 2 diabetes (T2D), T2D age diagnosed, T2D early onset, mean platelet volume, platelet count, white blood cell, HDL cholesterol, LDL cholesterol, triglycerides, total cholesterol, body mass index, waist hip ratio (BMI adjusted), waist circumference (BMI adjusted), height, percent fat mass, fasting glucose, fasting insulin, 2hr glucose, HbA1c, systolic blood pressure, diastolic blood pressure and QT interval.

Voight, B. F. et al. The metabochip, a custom genotyping array for genetic studies of metabolic, cardiovascular, and anthropometric traits. PLoS Genet 8, e1002793, doi:10.1371/journal.pgen.1002793 (2012).

ArrayCGH platforms used:

The Nimblegen arrays (135,000 probes across the genome) were used until the manufacturer stopped producing arrays, and we were required to move to a new platform. We then switched to Agilent arrays (180,000 probes), which provide similar coverage levels. We used both platforms on a subset of samples, and detected the same pattern of CNVs, including common (benign) and UCS CNVs. Full details of platforms used for each case are listed below:

aCGH platforms used for specific samples:
Nimblegen: TAK001, 2, 3, 4, 5, 6, 9, 10, 12, 13, 14, 15, 16, 17, 18, 19
Agilent: TAK002, 5, 7, 8, 11, 17, 19, 20, 21, 22, 23, 24, 25, 26, 27, 28
Samples analysed on both platforms: TAK002, 5, 17, 19

Supplementary Figure S1: Copy number variant interpretation algorithm.

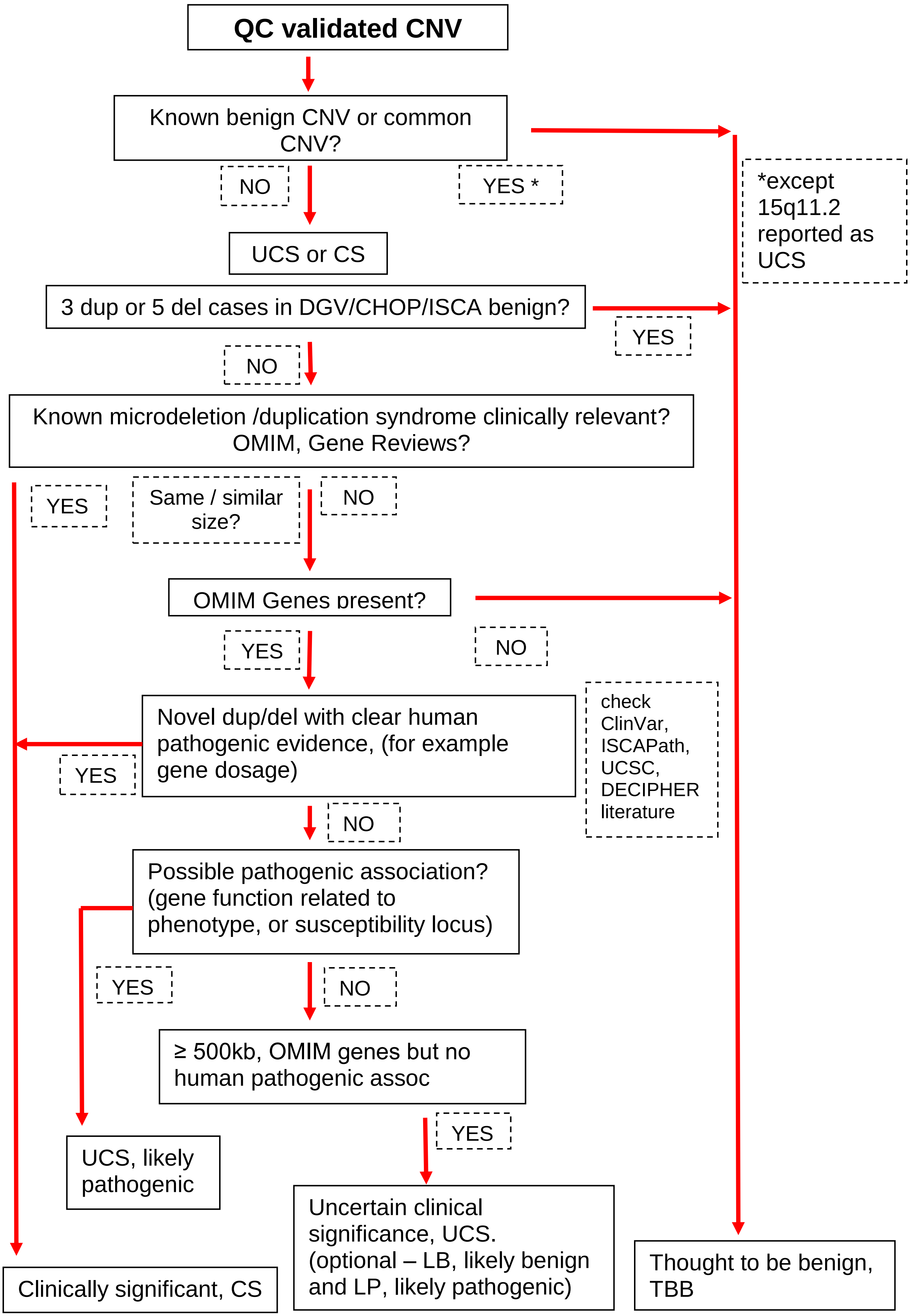

Supplementary Table S1: Complete list of CNVs detected in EqSCM cases in this study

Case	Hg19 coordinates	Call[1]	Location	CNV[2]	Size (kb)	Probes[3]	Gene content
EqSCM01	HG19 chr2:52,115,336-52,248,029	TBB	2p16.3	DEL	132	5	Agenic. Area of CNV.
	HG19 chr6:81,731,577-81,872,706	TBB	6q14.1	DUP	141	5	Agenic. Area of CNV.
	HG19 chr8:39,246,789-39,371,410	TBB	8p11.23	DUP	124	7	CNV 78
	HG19 chr14:22,562,556-22,953,081	TBB	14q11.2	DUP	390	38	CNV 62
	HG19 chr16:7,054,481-7,193,526	UCS	16p13.2	DEL	139	15	RBFOX1
EqSCM02							
	HG19 chr8:39,246,789-39,371,410	TBB	8p11.23	DUP	124	7	CNV 78
	HG19 chr14:22,562,556-22,959,329	TBB	14q11.2	DUP	396	39	CNV 62
	HG19 chrX:3,751,569-3,853,478	TBB	Xp22.33	DUP	101	12	CNV 82
EqSCM03							
NV1385O	HG19 chr8:39,246,789-39,394,134	TBB	8p11.23	DUP	147	8	CNV 78
	HG19 chr10:46,980,340-47,148,281	TBB	10q11.22	DUP	167	28	CNV 79
	HG19 chr17:44,194,591-44,275,529	TBB	17q21.31	DEL	80	14	CNV 91
	HG19 chr21:19,537,178-19,633,275	UCS	21q21.1	DEL	96	9	CHODL
	HG19 chr22:23,055,148-24,991,669	CS	22q11.22	DUP	1.94Mb	171	21 genes - from MIR650 to IGL.
	HG19 chrX:153,411,634-153,514,972	TBB	Xq28	DUP	103	9	CNV 83
EqSCM04	HG19 chr1:46,144,479-46,599,815	UCS	1p34.1	DUP	455	17	IPP, TMEM69, GPBP1L1, MAST1, PIK3R3.
	HG19 chr1:248,752,348-248,788,877	TBB	1q44	DUP	36	5	CNV 44
	HG19 chr14:22,562,556-22,959,329	TBB	14q11.2	DEL	396	39	CNV 62
	HG19 chr17:44,194,591-44,275,529	TBB	17q21.31	DEL	80	14	CNV 91
	HG19 chrX:3,751,569-3,853,478	TBB	Xp22.33	DUP	101	12	CNV 82
	HG19 chr19:20,617,028-20,714,335	TBB	19p12	DEL	97	7	Common deletion in DGV.
EqSCM05	HG19 chr10:65,998,732-66,193,916	TBB	10q21.3	DEL	195	7	Agenic. Area of CNV.
	HG19 chr14:22,477,962-22,943,320	TBB	14q11.2	DUP	465	42	CNV 62

	HG19 chrX:3,751,569-3,853,478	TBB	Xp22.33	DEL	101	12	CNV 82
	HG19 chrX:107,896,435-108,009,609	UCS	Xq22.3	DUP	113	9	IRS4, COL4A5.
EqSCM06	HG19 chr1:248,752,348-248,788,877	TBB	1q44	DUP	36	5	CNV 44
	HG19 chr3:46,799,630-46,849,443	TBB	3p21.31	DEL	48	5	CNV 84
	HG19 chr4:79,281,048-79,610,796	UCS	4q21.21	DUP	329	11	FRAS1, ANX3, LINC01094.
	HG19 chr8:39,246,789-39,371,410	TBB	8p11.23	DUP	124	7	CNV 78
	HG19 chr13:92,075,673-92,283,600	UCS	13q31.3	DEL	207	7	GPC5
	HG19 chr14:22,872,080-22,970,084	TBB	14p11.2	DUP	98	13	CNV62
	HG19 chrX:3,751,569-3,853,478	TBB	Xp22.33	DEL	101	12	CNV 82
	HG19 chrX:153,411,634-153,514,972	TBB	Xq28	DUP	103	9	CNV 83
EqSCM07	HG19 chr2:89,143,858-89,312,591	TBB	2p11.2	DUP	168	11	IGK. Common CNV.
	HG19 chr3:4,070,291-4,144,114	TBB	3p26.1	DUP	73	6	CNV 68
	HG19 chr3:162,514,534-162,619,142	TBB	3q26.1	DUP	104	6	Agenic. Common CNV.
	HG19 chr4:69,392,545-69,462,439	UCS-LB	4q13.2	DEL	69	6	UGT2B17
	HG19 chr8:15,952,011-16,021,745	TBB	8p22	DUP	69	7	MSR1. CNV9
	HG19 chr8:39,237,438-39,362,888	TBB	8p11.22	DUP	125	11	ADAM5P, ADAM3A. Common CNV.
	HG19 chr10:45,247,685-45,349,814	TBB	10q11.21	DEL	102	6	TMEM72-AS1. Common CNV.
	HG19 chr10:135,254,039-135,377,533	TBB	10q26.3	DUP	123	11	LOC619207, CYP2E1, SYCE1. Common CNV.
	HG19 chr11:55,377,910-55,450,789	TBB	11q11	DEL	70	8	Two copy loss. OR4P4, OR4S2, OR4C6. Common CNV.
	HG19 chr12:31,281,798-31,393,024	TBB	12p11.21	DUP	111	7	Possibly extends to DDX11. Common CNV.
	HG19 chr12:52,657,396-53,069,013	UCS	12q13.13	DUP	411	32	17 Keratin genes.
	HG19 chr14:19,376,762-20,414,233	TBB	14q11.2	DUP	1040	14	Common CNV.
	HG19 chr14:22,342,308-22,964,923	TBB	14q11.2	DEL	622	56	Common CNV.
	HG19 chr14:106,334,907-106,966,455	TBB	14q32.33	DUP	631	48	Common CNV.

	HG19 chr15:20,564,608-22,558,757	TBB	15q11.2-11.2	DUP	1990	33	Common CNV.
	HG19 chr22:23,056,562-23,245,889	TBB	22q11.22	DUP	189	27	Common CNV.
EqSCM08	HG19 chr1:149,041,933-149,243,968	TBB	1q21.2	DEL	202	15	NBPF25P. Common CNV.
	HG19 chr2:89,143,858-89,312,591	TBB	2p11.2	DUP	168	11	Common CNV.
	HG19 chr3:4,070,291-4,144,114	TBB	3p26.1	DUP	73	6	CNV 68
	HG19 chr3:162,514,534-162,619,142	TBB	3q26.1	DUP	104	6	Agenic. Common CNV.
	HG19 chr8:15,952,011-16,021,745	TBB	8p22	DUP	69	7	MSR1. CNV9
	HG19 chr8:19,375,578-19,426,577	UCS-LB	8p21.3	DEL	51	5	CSGALNACT1
	HG19 chr8:39,237,438-39,374,790	TBB	8p11.22	DUP	137	12	ADAM5P, ADAM3A. Common CNV.
	HG19 chr11:61,476,610-61,553,176	UCS-LB	11q12.2	DUP	76	8	DAGLA, C11orf9, DKFZP434K028.
	HG19 chr14:106,334,907-106,966,455	TBB	14q32.33	DUP	631	48	Common CNV.
	HG19 chr15:20,172,544-22,558,757	TBB	15q11.1-11.2	DUP	2390	37	Common CNV.
	HG19 chr17:44,171,888-44,345,039	TBB	17q21.31	DUP	173	12	Common CNV.
	HG19 chr22:23,056,562-23,245,889	TBB	22q11.22	DUP	189	27	Common CNV
	HG19 chr22:24,347,959-24,390,255	TBB	22q11.23	DUP	42	5	LOC391322, GSTT1, GSTTP2.
EqSCM09	HG19 chr1:248,752,348-248,788,877	TBB	1q44	DEL	36	5	CNV 44
	HG19 chr3:46,799,630-46,849,443	TBB	3p21.31	DEL	48	5	CNV 84
	HG19 chr4:131,956,494-132,348,098	TBB	4q28.3	DUP	391	13	Agenic. Common CNV.
EqSCM10	HG19 chr1:248,752,348-248,844,848	TBB	1q44	DEL	92	6	CNV 44
	HG19 chr8:39,246,789-39,371,410	TBB	8p11.23	DEL	124	7	CNV 78
	HG19 chr14:22,634,953-22,953,081	TBB	14q11.2	DUP	318	30	CNV 62
	HG19 chr17:44,185,649-44,267,524	TBB	17q21.31	DUP	81	14	CNV 81
	HG19 chr19:55,439,927-55,570,442	UCS	19q13.42	DUP	130	13	NLRP7, NLRP2, GP6, RDH13.

EqSCM11	HG19 chr2:44,519,680-44,543,829	TBB	2p21	DUP	24	7	SLC3A1. Common CNV.
	HG19 chr8:39,246,789-39,371,410	TBB	8p11.23	DEL	124	7	CNV 78
	HG19 chr10:46,980,161-47,140,244	TBB	10q11.22	DEL	160	7	Common CNV.
	HG19 chr14:106,335,742-106,732,386	TBB	14q32.33	DUP	396	6	Common CNV.
	HG19 chr22:22,008,249-22,189,094	UCS	22q11.21	DUP	180	51	PPL2, YPEL1, MAPK1.
	HG19 chrX:3,751,569-3,853,478	TBB	Xp22.33	DEL	84	10	CNV 82
	HG19 chrX:38,485,991-38,626,762	UCS	Xp11.4	DUP	140	15	TSPAN7
EqSCM12	HG19 chr8:8,108,863-8,142,313	TBB	8p23.1	DUP	33	6	Common CNV.
	HG19 chr8:39,246,789-39,371,410	TBB	8p11.23	DEL	124	7	Common CNV.
	HG19 chr14:22,465,154-22,970,084	TBB	14q11.2	DUP	504	47	CNV 62
	HG19 chr17:44,206,713-44,275,529	TBB	17q21.31	DUP	68	13	CNV 81
EqSCM13	HG19 chr8:39,246,789-39,371,410	TBB	8p11.23	DUP	124	7	CNV78
	HG19 chr14:22,374,537-22,970,084	TBB	14q11.2	DUP	595	53	CNV62
	HG19 chr17:44,206,713-44,275,529	TBB	17q21.31	DUP	68	13	CNV 81
	HG19 chrX:153,422,192-153,514,972	TBB	Xq28	DUP	92	5	CNV 83
EqSCM14	HG19 chr1:248,752,348-248,788,877	TBB	1q44	DEL	36	5	CNV 44
	HG19 chr9:209,254-254,239	TBB	9p24.3	DEL	44	6	DOCK8. Common CNV.
	HG19 chr10:46,980,340-47,148,281	TBB	10q11.22	DUP	167	28	CNV 79
	HG19 chr17:44,185,649-44,275,529	TBB	17q21.31	DUP	89	15	CNV 81
	HG19 chrX:3,763,818-3,853,478	TBB	Xp22.33	DEL	89	11	CNV 82
EqSCM15	HG19 chr2:242,864,903-243,006,028	TBB	2q37.3	DEL	141	15	CNV 52
	HG19 chr8:39,246,789-39,394,134	TBB	8p11.23	DUP	147	8	CNV 78
	HG19 chr10:83,506,502-83,585,097	UCS	10q23.1	DUP	78	7	Upstream of NRG3.
	HG19 chr17:44,210,699-44,275,529	TBB	17q21.31	DEL	64	12	CNV 81
EqSCM16	HG19 chr14:22,792,976-22,982,294	TBB	14q11.2	DUP	189	20	CNV 62

	HG19 chr17:44,206,713-44,275,529	TBB	17q21.31	DEL	68	13	CNV 81
	HG19 chrX:3,751,569-3,853,478	TBB	Xp22.33	DEL	101	12	CNV 82
EqSCM17	HG19 chr2:89,143,858-89,312,591	TBB	2p11.2	DUP	168	11	Agenic. Common CNV.
	HG19 chr3:4,070,291-4,144,114	TBB	3p26.1	DUP	73	6	CNV68
	HG19 chr4:69,392,545-69,462,439	TBB	4q13.2	DUP	69	6	UGT2B17. Common CNV.
	HG19 chr8:15,952,011-16,021,745	TBB	8p22	DEL	69	7	MSR1 CNV9
	HG19 chr8:39,237,438-39,362,888	TBB	8p11.22	DUP	125	11	ADAM5P, ADAM3A. Common CNV.
	HG19 chr10:135,254,039-135,377,533	TBB	10q26.3	DUP	123	11	LOC619207, CYP2E1, SYCE1. Common CNV.
	HG19 chr11:55,377,910-55,450,789	TBB	11q11	DEL	72	8	OR4P4, OR4S2, OR4C6. Common CNV.
	HG19 chr12:9,637,323-9,713,426	TBB	12p13.31	DUP	76	5	Possibly extends to DDX12P. Common CNV.
	HG19 chr13:70,008,862-70,183,894	TBB	13q21.33	DEL	175	7	Agenic.
	HG19 chr13:102,148,514-102,296,766	UCS	13q33.1	DEL	40	4	ITGBL1, within i1 (may encompass e1)
	HG19 chr13:102,148,514-102,296,767	UCS	13q33.2	DEL	50	5	ITGBL1, within i6 (may encompass e5)
	HG19 chr14:19,376,762-20,414,233	TBB	14q11.2	DUP	1040	14	Common CNV.
	HG19 chr14:22,342,308-22,964,923	TBB	14q11.2	DEL	622	56	Common CNV.
	HG19 chr14:106,334,907-106,966,455	TBB	14q32.33	DUP	631	48	Common CNV.
	HG19 chr15:20,481,702-22,558,757	TBB	15q11.1-11.2	DUP	2080	34	Common CNV.
	HG19 chr22:23,056,562-23,245,889	TBB	22q11.22	DUP	189	27	Common CNV.
	HG19 chrX:283,986-401,717	TBB	Xp22.33	DUP	117	18	Common CNV.
EqSCM18	HG19 chr12:31,356,144-31,399,162	TBB	12p11.21	DUP	43	8	Common CNV.
	HG19 chrX:3,751,569-3,799,236	TBB	Xp22.33	DEL	47	7	CNV82
EqSCM19	HG19 chr3:195,419,168-195,472,856	TBB	3q29	DEL	53	9	MIR570, MUC20. Common CNV.
	HG19 chr6:266,046-370,470	TBB	6p25.3	DUP	104	18	CNV72

	HG19 chr13:50,585,186-51,452,033	UCS	13q14.2	DEL	886	75	DLEU2, TRIM13, KCNRG, MIR16-1, MIR15A, DLEU1, ST13P4, DLEU7, RNASEH2B-AS1
	HG19 chr13:111,385,673-111,532,564	TBB	13q34	DUP	146	11	LINC00346, ANKRD10.
	HG19 chr14:22,509,226-22,953,081	TBB	14q11.2	DUP	443	42	CNV62
	HG19 chr14:106,246,283-106,906,968	TBB	14q32.33	DEL	660	25	Common CNV.
	HG19 chr15:20,432,851-22,332,238	TBB	15q11.1-11.2	DUP	1900	17	Common CNV.
	HG19 chr17:44,206,713-44,275,529	TBB	17q21.31	DEL	68	13	CNV 81
EqSCM20	HG19 chr1:149,041,933-149,243,968	TBB	1q21.2	DEL	170	7	NBPF25P. Common CNV.
	HG19 chr2:89,143,858-89,312,591	TBB	2p11.2	DUP	168	11	Agenic. Common CNV.
	HG19 chr3:4,070,291-4,144,114	TBB	3p26.1	DUP	73	6	Agenic. CNV 68
	HG19 chr3:195,419,168-195,459,590	TBB	3q29	DEL	40	8	MIR570, MUC20. Common CNV.
	HG19 chr8:15,952,011-16,021,745	TBB	8p22	DUP	69	7	MSR1. CNV9
	HG19 chr8:39,237,438-39,374,790	TBB	8p11.22	DUP	137	12	ADAM5P, ADAM3A Common CNV.
	HG19 chr10:45,247,685-45,349,814	TBB	10q11.21	DEL	102	6	TMEM72-AS1 Common CNV.
	HG19 chr14:106,334,907-106,966,455	TBB	14q32.33	DUP	631	48	Common CNV.
	HG19 chr15:20,432,851-22,558,757	TBB	15q11.2	DUP	2130	35	Common CNV.
	HG19 chr22:23,056,562-23,245,889	TBB	22q11.22	DUP	189	27	Common CNV.
EqSCM21	HG19 chr2:89,143,858-89,312,591	TBB	2p11.2	DUP	168	11	IGK. Common CNV.
	HG19 chr3:4,070,291-4,144,114	TBB	3p26.1	DUP	73	6	CNV 68
	HG19 chr3:162,514,534-162,619,142	TBB	3q26.1	DUP	104	6	Common CNV.
	HG19 chr3:195,419,168-195,472,856	TBB	3q29	DEL	53	9	MIR570, MUC20. Common CNV.
	HG19 chr4:69,392,545-69,462,439	UCS-LB	4q13.2	DUP	69	6	UGT2B17. Common CNV.
	HG19 chr7:142,825,843-142,890,669	TBB	7q34	DEL	64	6	PIP, TAS2R39. Common CNV.
	HG19 chr8:15,952,011-16,021,745	TBB	8p22	DUP	69	7	MSR1. CNV9

	HG19 chr8:39,237,438-39,374,790	TBB	8p11.22	DUP	137	12	ADAM5P, ADAM3A. Common CNV.
	HG19 chr11:55,377,910-55,450,789	TBB	11q11	DEL	72	8	OR4P4, OR4S2, OR4C6. Common CNV.
	HG19 chr12:86,925,372-87,027,515	TBB	12q21.32	DEL	102	9	MGAT4C, MIR548AL.
	HG19 chr14:22,360,671-22,952,280	TBB	14q11.2	DEL	591	53	CNV62
	HG19 chr14:106,334,907-106,966,455	TBB	14q32.33	DUP	631	48	Common CNV.
	HG19 chr15:20,603,042-22,558,757	TBB	15q11.1-11.2	DUP	1960	30	Common CNV.
	HG19 chr22:23,056,562-23,245,889	TBB	22q11.22	DUP	189	27	Common CNV.
	HG19 chr22:24,347,959-24,390,255	TBB	22q11.23	DUP	42	5	LOC391322, GSTT1, GSTTP2.
EqSCM22	HG19 chr1:149,041,933-149,224,044	TBB	1q21.2	DEL	182	14	NBPF25P. Common CNV.
	HG19 chr2:89,143,858-89,312,591	TBB	2p11.2	DUP	168	11	IGK. Common CNV.
	HG19 chr3:4,070,291-4,144,114	TBB	3p26.1	DUP	73	6	CNV 68
	HG19 chr3:162,514,534-162,619,142	TBB	3q26.1	DUP	104	5	Common CNV.
	HG19 chr3:195,419,168-195,472,856	TBB	3q29	DEL	53	9	MIR570, MUC20. Common CNV.
	HG19 chr4:69,392,545-69,462,439	UCS-LB	4q13.2	DUP	69	5	UGT2B17. Common CNV.
	HG19 chr8:15,952,011-16,021,745	TBB	8p22	DUP	69	7	MSR1. CNV9
	HG19 chr14:19,728,641-20,414,233	TBB	14q11.2	DUP	685	13	Common CNV.
	HG19 chr14:22,360,671-22,976,317	TBB	14q11.3	DEL	615	53	Common CNV.
	HG19 chr14:106,334,907-106,966,455	TBB	14q32.33	DUP	631	46	Common CNV.
	HG19 chr15:20,432,851-22,558,757	TBB	15q11.2	DUP	2130	35	Common CNV.
	HG19 chr22:23,056,562-23,245,889	TBB	22q11.22	DUP	189	26	Common CNV.
	HG19 chr22:24,347,959-24,390,255	TBB	22q11.23	DUP	42	5	LOC391322, GSTT1, GSTTP2.
EqSCM23	HG19 chr2:87,105,194-88,297,442	TBB	2p11.2	DUP	1190	13	LINC00152, MIR4435-1, MIR4435-2, PLGLB2, PLGLB1, RGPD1, RGPD2. Common CNV.
	HG19 chr2:89,143,858-89,312,591	TBB	2p11.2	DUP	168	11	IGK. Common CNV.
	HG19 chr3:4,070,291-4,144,114	TBB	3p26.1	DUP	73	6	CNV 68

	HG19 chr3:162,514,534-162,619,142	TBB	3q26.1	DUP	104	6	Common CNV.
	HG19 chr4:69,392,545-69,462,439	UCS-LB	4q13.2	DUP	69	6	UGT2B17. Common CNV.
	HG19 chr8:15,952,011-16,021,745	TBB	8p22	DUP	69	7	MSR1. CNV9
	HG19 chr8:39,237,438-39,374,790	TBB	8p11.22	DUP	137	12	ADAM5P, ADAM3A. Common CNV.
	HG19 chr10:45,247,685-45,349,814	TBB	10q11.21	DEL	102	6	TMEM72-AS1. Common CNV.
	HG19 chr10:46,984,913-47,148,547	TBB	10q11.22	DEL	163	13	Common CNV.
	HG19 chr14:19,376,762-20,414,233	TBB	14q11.2	DUP	1040	14	Common CNV.
	HG19 chr14:22,342,308-22,964,923	TBB	14q11.2	DEL	622	56	Common CNV.
	HG19 chr14:106,334,907-106,966,455	TBB	14q32.33	DUP	631	48	Common CNV.
	HG19 chr17:44,197,448-44,345,039	TBB	17q21.31	DUP	147	10	Common CNV.
	HG19 chr22:23,056,562-23,245,889	TBB	22q11.22	DUP	189	27	Common CNV.
EqSCM24	HG19 chr1:149,041,933-149,209,290	TBB	1q21.2	DEL	167	13	NBPF25P. Common CNV.
	HG19 chr2:89,143,858-89,312,591	TBB	2p11.2	DUP	168	11	IGK. Common CNV.
	HG19 chr3:4,070,291-4,144,114	TBB	3p26.1	DUP	73	6	CNV 68
	HG19 chr3:162,514,534-162,619,142	TBB	3q26.1	DUP	104	5	Common CNV.
	HG19 chr3:195,419,168-195,457,766	TBB	3q29	DEL	38	7	MIR570, MUC20. Common CNV.
	HG19 chr4:69,392,545-69,462,439	UCS-LB	4q13.2	DUP	69	5	UGT2B17. Common CNV.
	HG19 chr8:15,952,011-16,021,745	TBB	8p22	DUP	69	7	MSR1. CNV9
	HG19 chr8:39,237,438-39,362,888	TBB	10q26.3	DUP	125	11	LOC619207, CYP2E1, SYCE1 Common CNV.
	HG19 chr10:45,247,685-45,349,814	TBB	10q11.21	DEL	102	6	TMEM72-AS1. Common CNV.
	HG19 chr10:46,976,157-47,148,547	TBB	10q11.22	DUP	172	14	Common CNV.
	HG19 chr11:134,353,814-134,711,673	TBB	11q25	DUP	357	19	LOC283177. Common CNV.
	HG19 chr14:106,334,907-106,966,455	TBB	14q32.33	DUP	631	46	Common CNV.
	HG19 chr15:20,603,042-22,558,757	TBB	15q11.1-11.2	DUP	1960	30	Common CNV.
	HG19 chr22:23,056,562-23,245,889	TBB	22q11.22	DUP	189	26	Common CNV.

	HG19 chr22:24,347,959-24,390,255	TBB	22q11.23	DUP	42	5	LOC391322, GSTT1, GSTTP2.
EqSCM25	HG19 chr2:89,143,858-89,312,591	TBB	2p11.2	DUP	168	11	IGK. Common CNV.
	HG19 chr3:4,070,291-4,144,114	TBB	3p26.1	DUP	73	6	CNV 68
	HG19 chr3:162,514,534-162,619,142	TBB	3q26.1	DUP	104	5	Common CNV.
	HG19 chr3:195,419,168-195,444,194	TBB	3q29	DEL	25	5	LINC00969, MIR570.
	HG19 chr8:15,952,011-16,021,745	TBB	8p22	DUP	69	7	MSR1. CNV9
	HG19 chr8:39,237,438-39,362,888	TBB	8p11.22	DUP	125	11	ADAM5P, ADAM3A. Common CNV.
	HG19 chr10:45,247,685-45,349,814	TBB	10q11.21	DEL	102	6	TMEM72-AS1. Common CNV.
	HG19 chr11:55,377,910-55,450,789	TBB	11q11	DEL	72	8	OR4P4, OR4S2, OR4C6. Common CNV.
	HG19 chr11:134,353,814-134,711,673	TBB	11q25	DUP	357	19	LOC283177. Common CNV.
	HG19 chr12:9,637,323-9,713,426	TBB	12p13.31	DUP	76	5	Possibly extends to DDX12P. Common CNV.
	HG19 chr14:19,728,641-20,414,233	TBB	14q11.2	DUP	685	13	Common CNV.
	HG19 chr14:22,360,671-22,976,317	TBB	14q11.3	DEL	615	53	Common CNV.
	HG19 chr14:104,793,823-104,936,775	TBB	14q32.33	DUP	142	9	Common CNV.
	HG19 chr14:106,334,907-106,966,455	TBB	14q32.33	DUP	631	46	Common CNV.
	HG19 chr15:32,021,733-32,510,864	TBB/UCS-LB	15q13.3	DUP	489	27	CHRNA7
	HG19 chr19:20,607,860-20,701,621	TBB	19p12	DEL	93	5	ZNF826P. Common CNV.
	HG19 chr22:23,056,562-23,245,889	TBB	22q11.22	DUP	189	26	Common CNV.
	HG19 chr22:24,347,959-24,390,255	TBB	22q11.23	DUP	42	5	LOC391322, GSTT1, GSTTP2.
EqSCM26	HG19 chr1:248,727,929-248,785,563	TBB	1q44	DEL	57	6	CNV 44
	HG19 chr2:37,959,707-37,994,075	TBB	2p22.2	DUP	34	5	Common CNV.
	HG19 chr2:89,143,858-89,312,591	TBB	2p11.2	DUP	168	11	IGK. Common CNV.
	HG19 chr3:4,070,291-4,144,114	TBB	3p26.1	DUP	73	6	CNV 68
	HG19 chr3:195,419,168-195,472,856	TBB	3q29	DEL	53	9	MIR570, MUC20. Common CNV.

	HG19 chr8:15,952,011-16,021,745	TBB	8p22	DUP	69	7	MSR1. CNV9
	HG19 chr8:39,237,438-39,374,790	TBB	8p11.22	DUP	137	12	ADAM5P, ADAM3A. Common CNV.
	HG19 chr10:45,247,685-45,349,814	TBB	10q11.21	DEL	102	6	TMEM72-AS1. Common CNV.
	HG19 chr12:9,637,323-9,713,426	TBB	12p13.31	DUP	76	5	May extend to DDX12P. Common CNV.
	HG19 chr14:22,368,864-22,976,317	TBB	14q11.2	DEL	607	52	Common CNV.
	HG19 chr14:106,334,907-106,966,455	TBB	14q32.33	DUP	631	46	Common CNV.
	HG19 chr15:20,172,544-22,558,757	TBB	15q11.1-11.2	DUP	2390	37	Common CNV.
	HG19 chr16:21,599,687-21,951,439	TBB	16p12.2	DEL	351	13	IGSF6, OTOA, METTL9, RRN3P1. Common CNV.
	HG19 chr17:44,171,888-44,345,039	TBB	17q21.31	DUP	173	12	Common CNV.
	HG19 chr22:23,056,562-23,245,889	TBB	22q11.22	DUP	189	26	Common CNV.
EqSCM27	HG19 chr1:16,840,487-17,231,818	TBB	1p36.13	DEL	391	10	MIR3675, NBPF1, CROCCP2, MST1P2, MIR3675, ESPNP, MST1P9, MIR3675. Common CNV.
	HG19 chr2:89,143,858-89,312,591	TBB	2p11.2	DUP	168	11	IGK. Common CNV.
	HG19 chr3:4,070,291-4,144,114	TBB	3p26.1	DUP	73	6	CNV 68
	HG19 chr3:195,419,168-195,459,590	TBB	3q29	DEL	40	8	MIR570, MUC20. Common CNV.
	HG19 chr7:110,744,611-110,849,681	UCS	7q31.1	DEL	105	10	IMMP2L, LRRN3.
	HG19 chr8:15,952,011-16,021,745	TBB	8p22	DUP	69	7	MSR1. CNV9
	HG19 chr8:39,237,438-39,362,888	TBB	8p11.22	DUP	125	11	ADAM5P, ADAM3A. Common CNV.
	HG19 chr14:19,728,641-20,414,233	TBB	14q11.2	DUP	685	13	Common CNV.
	HG19 chr14:106,334,907-106,966,455	TBB	14q32.33	DUP	631	48	Common CNV.
	HG19 chr15:20,481,702-22,558,757	TBB	15q11.1-11.2	DUP	2080	34	Common CNV.
	HG19 chr22:23,056,562-23,245,889	TBB	22q11.22	DUP	189	27	Common CNV.
	HG19 chr22:24,347,959-24,390,255	TBB	22q11.23	DUP	42	5	LOC391322, GSTT1, GSTTP2.
EqSCM28	HG19 chr6:266,079-375,950	TBB	6p25.3	DEL	109	19	Common CNV.
	HG19 chr8:39,246,720-39,371,396	TBB	8p11.22	DEL	124	7	ADAM5P, ADAM3A. Common CNV.

HG19 chr9:398,958-561,122	TBB UCS-LB	9p24.3	DUP	162	17	DOCK8, KANK1. Area of CNV.
HG19 chr10:46,980,161-47,140,244	TBB	10q11.22	DEL	160	7	Common CNV.
HG19 chr14:106,335,742-106,732,386	TBB	14q32.33	DUP	396	6	Common CNV.
HG19 chr14:106,921,691-107,208,511	TBB	14q32.34	DUP	286	12	Common CNV.

[1] Call (interpretation of CNV functional significance – refer Supp. Fig. S1). CS, Clinically significant; TBB, Thought to be benign; UCS, Uncertain clinical significance; UCS–LB, UCS likely benign; UCS-LP, UCS likely pathogenic.
[2] Nature of CNV. Duplication (dup) or deletion (del).
[3] Number of arrayCGH probes generating the signal

Supplementary Table S2: Exome read count and coverage metrics

Sample	TARGET_TERRITORY	TOTAL_READS	MEAN_TARGET_COVERAGE	TARGET_BASES_10X	TARGET_BASES_20X	TARGET_BASES_30X
TAK001	62,085,286	19,440,510	14.52	0.67	0.27	0.06
TAK002	62,085,286	23,259,954	17.70	0.73	0.41	0.15
TAK003	62,085,286	26,280,648	19.85	0.76	0.48	0.21
TAK004	62,085,286	24,470,786	18.30	0.73	0.43	0.17
TAK005	62,085,286	17,249,925	13.16	0.62	0.22	0.04
TAK006	62,085,286	19,741,681	14.83	0.66	0.31	0.08
TAK007	62,085,286	18,084,914	13.71	0.64	0.24	0.05
TAK008	62,085,286	18,509,610	14.15	0.66	0.26	0.06
TAK009	62,085,286	19,986,364	15.30	0.68	0.31	0.08
TAK010	62,085,286	19,775,844	14.83	0.68	0.28	0.07
TAK011	62,085,286	20,101,353	15.23	0.69	0.30	0.07
TAK012	62,085,286	16,932,783	13.00	0.61	0.22	0.04
TAK013	62,085,286	26,260,934	19.08	0.74	0.40	0.17
TAK014	62,085,286	30,737,799	20.62	0.77	0.45	0.21
TAK015	62,085,286	24,568,099	16.88	0.70	0.33	0.12
TAK016	62,085,286	23,365,465	16.71	0.69	0.32	0.12
TAK017	62,085,286	19,823,276	14.31	0.63	0.24	0.08
TAK018	62,085,286	26,818,236	19.46	0.75	0.41	0.18
TAK019	62,085,286	22,463,554	16.28	0.68	0.31	0.11
TAK020	62,085,286	24,225,495	17.69	0.72	0.36	0.14
TAK021	62,085,286	26,375,671	18.97	0.74	0.40	0.17
TAK022	62,085,286	25,536,206	18.61	0.73	0.39	0.16
TAK023	62,085,286	23,748,264	17.25	0.71	0.34	0.13
TAK024	62,085,286	27,887,246	20.22	0.76	0.44	0.20
min		16,932,783	13.00	0.61	0.22	0.04
mean		22,735,192	16.69	0.70	0.34	0.12
max		30,737,799	20.62	0.77	0.48	0.21
SD		3,642,944	2.31	0.05	0.08	0.05
